# Reticulated, Hyperchromic Rash in a Striated Pattern Mimicking Atopic Dermatitis and Fungal Infection in a 2-Month-Old Female: A Case of Incontinentia Pigmenti

**DOI:** 10.1155/2016/9512627

**Published:** 2016-04-19

**Authors:** Nina Poliak, Alexandre Le, Anthony Rainey

**Affiliations:** ^1^The Wright Center for Graduate Medical Education, Scranton, PA 18510, USA; ^2^Department of Pediatrics, The Commonwealth Medical College, Scranton, PA 18509, USA

## Abstract

We present a 12-month-old Hispanic female with a reticulated, hyperchromic rash in a striated pattern appearing on upper and lower extremities and trunk and back since the age of 6 weeks. Over the next 10 months, the rash persisted. The rash did not respond to treatment with antifungals and steroids. During her 6-month wellness visit, the patient was diagnosed with incontinentia pigmenti (IP), a rare X-linked dominant disorder, fatal to male fetuses in utero. IP can lead to serious neurological and ophthalmologic consequences. Early diagnosis by primary care physicians and parental education about the condition are essential for prevention of retinal detachment, developmental delay, and dental abnormalities.

## 1. Introduction

Incontinentia pigmenti (IP) is an X-linked dominant disorder with prevalence of 1 : 500,000 and incidence of 0.0025% of live births. The disease affects the skin, central nervous system, eyes, and skeletal system [1–3]. It results from mutations of the* IKBKG* gene on the X-chromosome which causes a loss of function of the nuclear factor-kappa B (*NF-κB*), leaving cells susceptible to apoptosis from intrinsic factors [2, 3]. Patients with IP are 97% female. The disease leads to death in utero in males homozygous for the mutation with a typical karyotype, but males with Klinefelter syndrome may develop IP [4]. Cutaneous and central nervous system manifestations of IP vary depending on lyonization of the X-chromosome [5]. Cutaneous lesions follow the lines of Blaschko and appear by the age of 6 weeks in 96% of cases [1]. Among patients with IP, 36.5% have detectable eye pathology and 60 to 90% of those have retinal issues [3].

The lesions of incontinentia pigmenti follow four chronologic phases, which make up the major criteria for diagnosis [6]. It is uncommon for all stages to be seen in the same case [7].

- Phase 1, also called the inflammatory or vesicular phase, consists of largely papular lesions with scattered vesicles which can become pustular and then bullous. It appears at birth and can persist for months. Biopsy of blisters in the vesicular stage shows epidermal vesicles filled with eosinophils in 74% of cases [1, 4, 6]. This phase can be confused with herpes simplex or impetigo [6].
- Phase 2, the verrucous phase, consists of irregular, linear, warty papules on one or more extremities (hands and feet) [1]. It usually appears within two to six weeks and usually disappears by six months of age [6].
- Phase 3, the pigmentary phase, consists of thin bands of slate-brown to blue-gray coloration in lines and swirls on the extremities and trunk. It is sometimes purpuric at onset and progresses until 2 years of age, stabilizes, persists for years, fades gradually, and disappears by adolescence in two-thirds of patients. At this stage, biopsy would reveal incontinence of skin pigment.
- Phase 4 or the hypopigmented or atrophic phase consists of atrophic streaks on arms, thighs, trunks, and calves with decreased hair, eccrine glands, and sweat pores of affected children.

Other minor diagnostic criteria include palate anomalies, nipple and breast anomalies, multiple male miscarriages, and typical skin pathohistological findings [2].

Signs of incontinentia pigmenti also include cicatricial alopecia, nail dystrophy, dental abnormalities (delayed dentition, partial anodontia, and pegged or conical teeth), neurologic abnormalities (seizures, developmental delay, and spastic abnormalities), ophthalmic changes (strabismus, cataracts, optic atrophy, retinal neovascularization or detachment, and bilateral blindness), cardiac anomalies, skeletal malformations (microcephaly, syndactyly, supernumerary ribs, hemiatrophy, and shortening of arms and legs), and subungual tumors [1]. Skin lesions are subtle and are best viewed with side lighting or Wood's lamp [1]. Lesions are seen during adolescence and may remain permanently. Biopsy shows no eccrine glands and no pigment in the epidermis [6].

## 2. Case Presentation

We present the case of a 12-month-old female who was evaluated at the pediatric clinic in rural Northeastern Pennsylvania, United States. The patient was a product of a full term pregnancy, born vaginally after an uncomplicated pregnancy from nonconsanguineous, phenotypically healthy parents. However, the patient's mother was noted to have a history of multiple spontaneous abortions prior to this pregnancy.

Initially, at the age of 6 weeks, the patient was seen for a rash on arms and legs, between body folds, which was refractory to the application of moisturizing skin lotion. On that visit, she was diagnosed with a fungal infection and was prescribed nystatin. Unfortunately, the rash persisted despite the use of nystatin and over-the-counter petroleum jelly on both lower extremities. This demonstrates how IP can be confused for other dermatologic diseases [4].

At her 2-month wellness visit, the patient demonstrated the same rash. The rash was reticulated, hyperchromic, in a striated pattern, appearing bilaterally on both lower extremities, and extending into the gluteal area. It also appeared on both upper extremities. Therefore, the patient was referred to a pediatric dermatologist for evaluation of possible autoimmune disease. At the age of 3 months, the rash appeared as linear hyperpigmented areas in a swirled pattern on legs, chest, and arms. Subsequently, at the age of 5 months, there was brown, swirled hyperpigmentation on the trunk, legs, chest, and arms along with pigmentation resembling calligraphy on the extremities and chin. The patient was therefore diagnosed with a mosaic pigment anomaly and was prescribed the topical glucocorticoids fluocinonide and mometasone to no effect.

**Figure 1:**
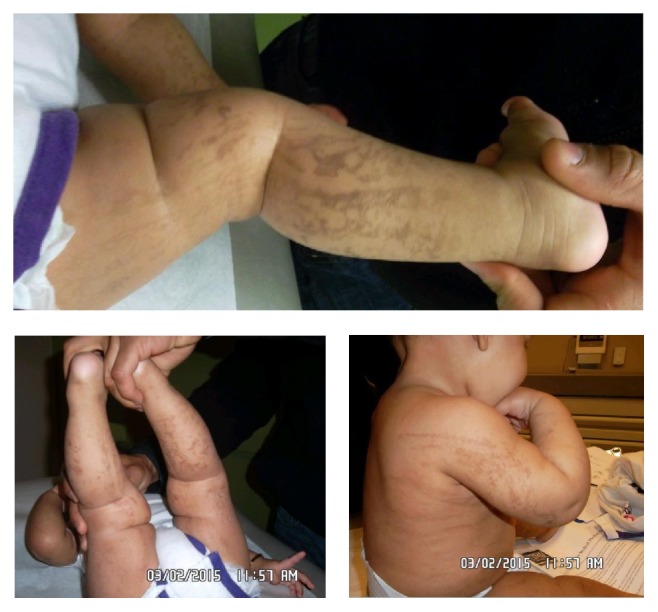
Hyperpigmented lesions in swirls on both the upper and lower extremities during 6-month exam.

At 6 months of age, the same pattern of hyperpigmentation was noted in the upper and lower extremities bilaterally (Figure 1). At that point, the patient was diagnosed with incontinentia pigmenti.

For surveillance purposes, the patient was referred to a pediatric neurologist for evaluation. The patient was also seen by a pediatric ophthalmologist for retinal evaluation and by a pediatric dentist for assessment of dental development. Parents were educated about the disease and were advised that no medical treatment was necessary for the rash and all topical steroids were discontinued. It was explained that the diagnosis could be made with confidence from clinical features. Confirmatory skin biopsy and genetic testing for the* IKBKG* gene were declined. Since that visit, an initial eye exam was performed at the age of 10 months and was found to be normal for age, without evidence of retinal detachment.

**Figure 2:**
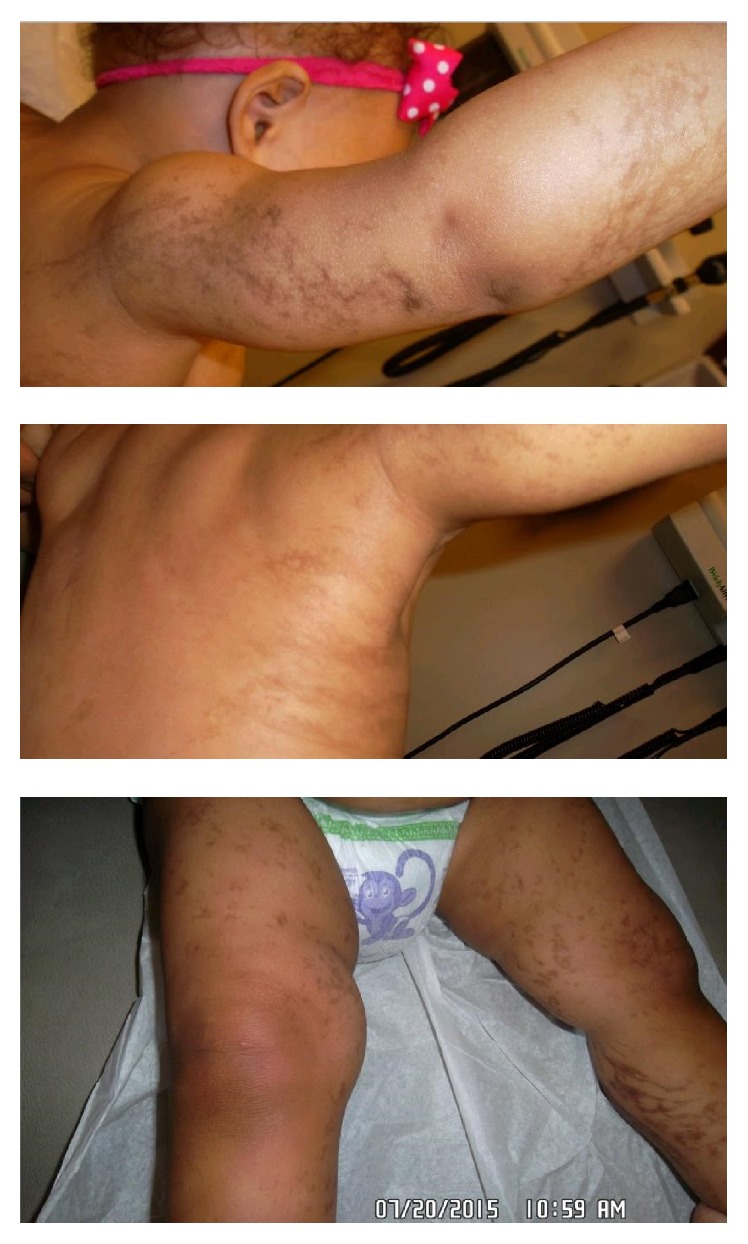
Hyperpigmented lesions on both upper and lower extremities at 12-month visit.

**Figure 3:**
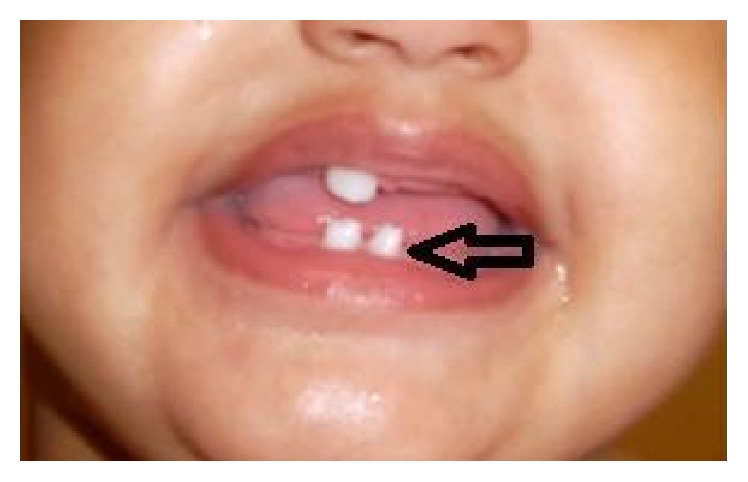
Pegged tooth (lower central incisor) seen at 12-month visit.

The patient returned to the clinic at the age of 12 months. The patient was free of sequelae from prolonged unnecessary corticosteroid therapy. The hyperpigmented skin pattern was still present on both of her upper and lower extremities (Figure 2). In addition, a pegged tooth was seen at the level of the lower central incisor (Figure 3). Therefore, the importance of following up with the pediatric dentist was stressed.

## 3. Discussion

Our 12-month-old patient presented to her primary care physician at the age of 6 weeks with phase 3 of incontinentia pigmenti (IP). She met one major and one minor criteria for diagnosis, both of which were clinical in nature. The major criterion was the appearance of the hyperpigmented rash in swirls on both upper and lower extremities, which persisted all the way to her 12-month wellness visit and which are expected to disappear by adolescence. The minor criterion was the conical appearance of her lower central incisor, for which parents were advised to seek a dental evaluation. Our patient did not display all the cutaneous stages.

Management of IP consists of early intervention, including serial ophthalmologic examinations for retinal involvement, neurological and neuropsychological evaluation for seizures and evidence of developmental delay along with baseline brain MRI with and without contrast at ages 3 to 6 months, and dental evaluation by age 2 [1, 8]. Skin lesions usually clear spontaneously and do not necessitate any treatment [1]. Nonetheless, a study by Poziomczyk et al., in 2014, showed that a combination of topical steroids (diflucortolone valerate and chlorquinaldol) led to rapid improvement of inflammatory lesions [6]. If the patient is diagnosed at birth through preimplantation genetic testing, a dilated fundus exam and a fluorescein angiogram are recommended before the neonate leaves the hospital. If normal, follow-up eye exams and dilation should take place monthly until the age of four months, every three months from ages four months to one year, every six months from ages one to three years, and annually thereafter. Most patients have normal vision and have similar prevalence of near and far-sightedness as the general population [8]. Dental anomalies can influence the patients' quality of life but usually do not necessitate urgent interventions, except in cases of cleft lip or cleft palate [9].

It is also important to obtain a history of cutaneous disorder during the neonatal or infantile period. As this case shows, history and physical exam of the patient's mother for subtle signs are important. Indeed, mothers with cicatricial alopecia, conical incisors, nail dystrophy, or atrophic skin streaks are 50% likely to have an affected daughter and have 50% chance of aborting a male fetus. In these cases, for future pregnancies, preimplantation genetic diagnosis is possible [1]. These techniques may become important during our patient's childbearing years.

## 4. Conclusion

Our case highlights a rare instance of diagnosis and appropriate follow-up of IP in a rural setting. Early recognition and diagnosis of this rare disease by general practitioners is essential, regardless of the patient and physician's geographical background. Timely diagnosis allows early surveillance steps such as ophthalmologic, neurologic, dental examinations, and genetic testing in order to prevent complications. The relevance of these points is reflected in NIH guidelines, which suggest monthly eye exams before the age of 4 months [10], as most serious ocular injuries involve vascularization of the retina in the first few months of life [6]. It is also imperative to educate general practitioners not to confuse IP with other skin conditions. Confusion with atopic dermatitis can lead to long-term use of high-dose topical steroids and subsequent serious side effects. Finally, parental education, discussion of preventative care, and family planning are extremely important for optimal long-term prognosis. Overall, astute diagnosis and early management of IP lead to ideal outcome for patients.
